# Defining the hypoxic thresholds that trigger blood-brain barrier disruption: the effect of age

**DOI:** 10.18632/aging.206241

**Published:** 2025-05-01

**Authors:** Arjun Sapkota, Sebok K. Halder, Richard Milner

**Affiliations:** 1 San Diego Biomedical Research Institute, San Diego, CA 92121, USA

**Keywords:** blood-brain barrier integrity, endothelial, proliferation, microglia, chronic mild hypoxia, hypoxic threshold

## Abstract

Chronic mild hypoxia (CMH; 8% O_2_) triggers transient blood-brain barrier (BBB) disruption, an effect greatly increased with age. As BBB disruption predisposes to neuronal death and cognitive decline, here we defined the hypoxic thresholds that trigger BBB breakdown in young and aged mice, and then defined the age at which hypoxia-induced BBB disruption significantly increases. Dual-immunofluorescence of brain sections demonstrated that the thresholds required to trigger hypoxia-induced BBB disruption (CD31/fibrinogen) and endothelial proliferation (CD31/Ki67) were much lower in aged mice (15% O_2_) compared to young (13% O_2_). Hypoxia-induced endothelial proliferation was relatively constant across the age range, but advanced age strongly enhanced the degree of BBB disruption (4-6-fold greater in 23 months vs. 2 months old). While the BBB became more vulnerable to hypoxic disruption at 12–15 months, a large step-up also occurred at the surprisingly young age 2–6 months. Our data demonstrates that the aged BBB is far more sensitive to hypoxia-induced BBB disruption than the young and define the hypoxic thresholds that trigger hypoxia-induced BBB disruption in young and aged mice. This information has translational implications for people exposed to hypoxia and for those living with hypoxia-associated conditions such as asthma, emphysema, ischemic heart disease, and apnea.

## INTRODUCTION

We recently demonstrated that exposure to chronic mild hypoxia (CMH; 8% O_2_) in young (2 months old) mice triggers a cerebrovascular remodeling response that includes endothelial proliferation and low levels of transient blood-brain barrier (BBB) disruption that is accompanied by microglial activation and aggregation around leaky blood vessels [1]. Strikingly, the extent of hypoxia-induced BBB disruption is greatly amplified (5-10-fold) in aged (20 months old) mice [2]. As hypoxia is a common component of many age-related diseases including chronic obstructive pulmonary disease (COPD), asthma, ischemic heart disease, heart failure, and sleep apnea, it follows that in the elderly population, hypoxic events could trigger BBB breakdown, culminating in neuronal dysfunction, neurodegeneration, and vascular dementia [3–6]. Consistent with this idea, several studies have demonstrated increased dementia risk in people who suffer from hypoxia-inducing conditions such as sleep apnea and COPD [3–6]. Further support is provided by our recent finding that when aged mice are exposed to CMH, they manifest marked BBB disruption, resulting in neuronal loss and decline in cognitive function [7].

Considering the pathogenic potential of the link between hypoxic exposure, BBB disruption, neuronal loss, and cognitive decline, and the notion that this could affect a large number of (particularly aged) people, it becomes a high priority to dig deeper into this connection to address some important fundamental questions. First, what hypoxic level is sufficient to trigger vascular remodeling and BBB breakdown, and how does age influence the hypoxic threshold that triggers BBB disruption? Second, at what age do cerebral blood vessels become more susceptible to hypoxia-induced disruption? In this study, we addressed these fundamental questions by first exposing young (2 months old) and aged (20 months old) mice to a range of oxygen levels from normoxia (21% O_2_) to marked hypoxia (8% O_2_) to define the hypoxic thresholds that triggers vascular remodeling and BBB disruption at the two different ages. Next, we investigated at what specific age mice become more susceptible to hypoxia, by comparing mice of 8 different ages (from 2 months to 23 months) to a fixed (8% O_2_) level of hypoxia. In both types of study, we evaluated the impact of hypoxia on the following parameters: endothelial proliferation (an early event in angiogenesis) and BBB disruption, and because our previous work highlights a close connection between BBB disruption and microglial activation, we also examined microglial activation and proliferation.

## RESULTS

### Hypoxia-induced cerebrovascular remodeling is triggered at a higher O_2_ level in aged mice

The main goal of these studies was to define the hypoxic threshold that triggers cerebral blood vessels to remodel. We evaluated endothelial proliferation as this is one of the earliest stages of angiogenesis, and BBB disruption, which occurs as a transient, but potentially pathogenic, side-effect of the vascular remodeling process [1, 2, 8]. Young (2 months old) and aged (20 months old) female C57BL6/J mice were maintained for 4 days under control conditions (normoxia; 21% O_2_) or at different levels of hypoxia, including 14, 13, 12, 10, and 8% O_2_. In prior studies we demonstrated that hypoxic-induced vascular remodeling occurs in all areas of the brain, with the strongest effect observed in the midbrain and olfactory bulb [1, 2]; therefore, most of the analyses in the current study were performed in the midbrain. A 4-day incubation period was selected because this is the time-point at which many of the remodeling parameters show peak levels, including endothelial proliferation, and fibronectin and α5β1 integrin expression [8].

**Figure 1:**
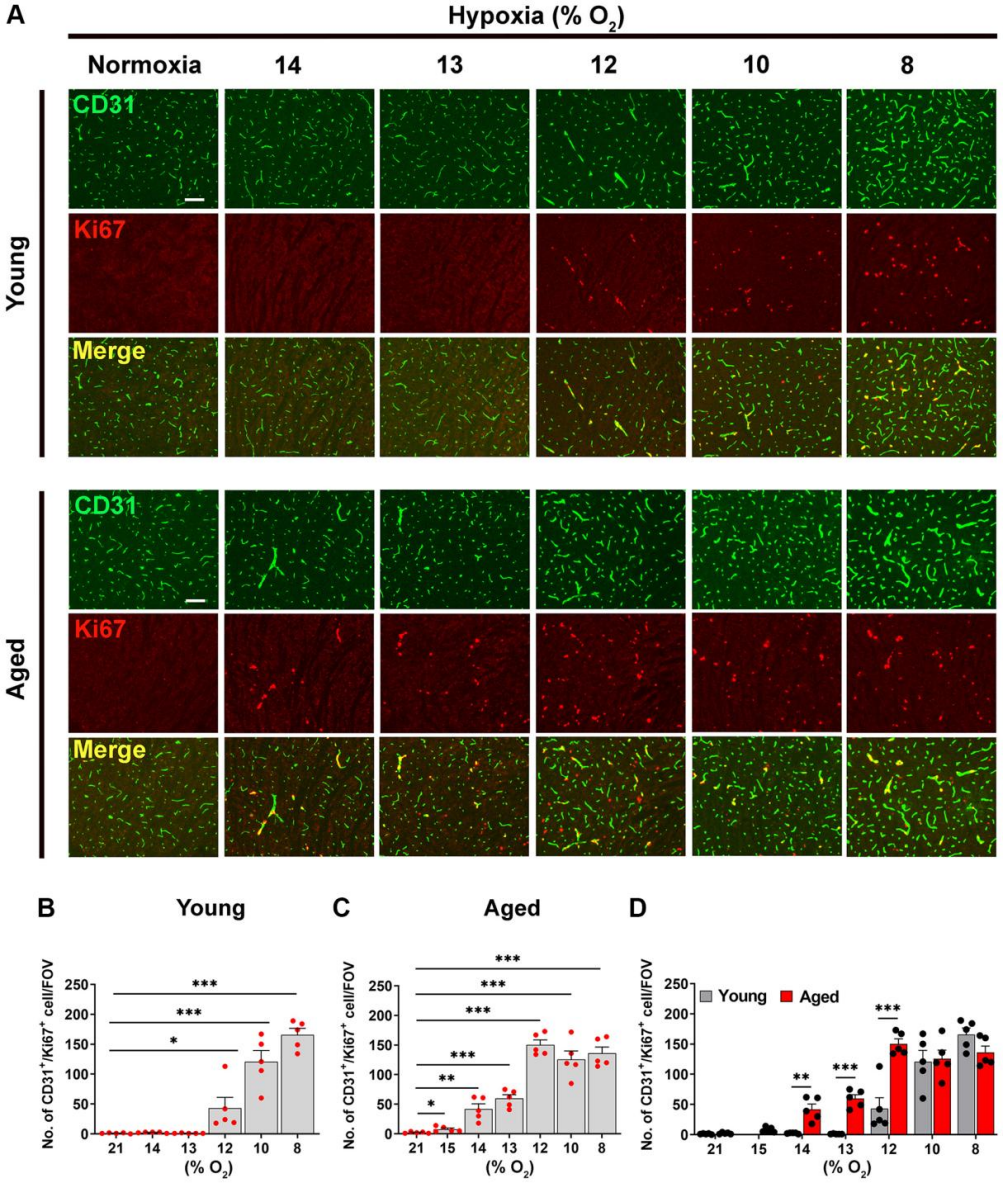
**Hypoxia-induced cerebrovascular remodeling is triggered at a higher O_2_ level in aged mice.** (**A**) Frozen brain sections taken from young (2 months) and aged (20 months) mice exposed to normoxia or different levels of hypoxia (15–8% O_2_) for 4 days were stained for the endothelial marker CD31 (AlexaFluor-488) and Ki67 (Cy-3). Images were captured in the midbrain. Scale bars = 100 μm. (**B**–**D**) Quantification of the density of CD31^+^/Ki67^+^ cells following normoxia or 4 days exposure to the different levels of hypoxia in young (**B**), aged (**C**) and both combined (**D**). Results are expressed as the mean ± standard error of the mean (SEM) (*n* = 5 mice/group). ^*^p < 0.05, ^**^p < 0.01, ^***^p < 0.001. One-way analysis of variance (ANOVA) followed by Tukey’s multiple comparison post-hoc test. Note that aged mice are more sensitive to the effects of hypoxia in that they display endothelial proliferation at a higher O_2_ level than young mice.

As one of the earliest and most sensitive markers of angiogenesis is endothelial proliferation, we performed dual-immunofluorescence (dual-IF) on frozen brain sections with the endothelial cell marker CD31 and the proliferation marker Ki67 to quantify endothelial proliferation. Consistent with previous studies [1, 2, 8], this showed that the young normoxic brain had a total absence of proliferating endothelial cells (Figure 1A, upper panel). In these young brains, significant levels of endothelial cell proliferation (identified by CD31/Ki67 dual-positive cells) were not observed until ambient O_2_ levels were reduced to 12% or lower (Figure 1A, upper panel), at which point endothelial proliferation increased in a tight dose-response manner as the level of hypoxic stimulus was increased from 12% down to 8% O_2._ (Figure 1B). The aged brain also showed no sign of endothelial proliferation under normoxic conditions (Figure 1A, lower panel). However, compared to young brain, the aged brain appeared to be more sensitive to the effect of hypoxia in that endothelial proliferation was first observed when O_2_ levels were reduced to the milder hypoxia level of 14% or lower (Figure 1A, lower panel). Because 14% O_2_ triggered endothelial proliferation in the aged mouse brain, we introduced an even milder level of 15% O_2_ for studying aged mice, and this also triggered a small but significant degree of endothelial proliferation Figure 1C). Quantification of these studies indicates that compared to young (2-month-old) mice, the brains of aged (20 months) mice are more sensitive to the effects of hypoxia, with the hypoxic threshold that promotes endothelial proliferation defined as 12% O_2_ in young mice but 15% O_2_ in aged mice (Figure 1B, 1C). This age-related difference in responsiveness to hypoxia is well illustrated in Figure 1D, which shows that the rates of endothelial proliferation are similar for young and aged mice at severe hypoxic levels of 10 or 8% O_2,_ but at the less severe level of 12% O_2_, while the rate of endothelial proliferation in young mice had fallen to less than a third of the maximum rate seen at 8% O_2_, that in aged mice at 12% O_2_ was maintained equal to that seen at 10 and 8% O_2._

### Aged mice are more susceptible to hypoxia-induced BBB disruption

**Figure 2:**
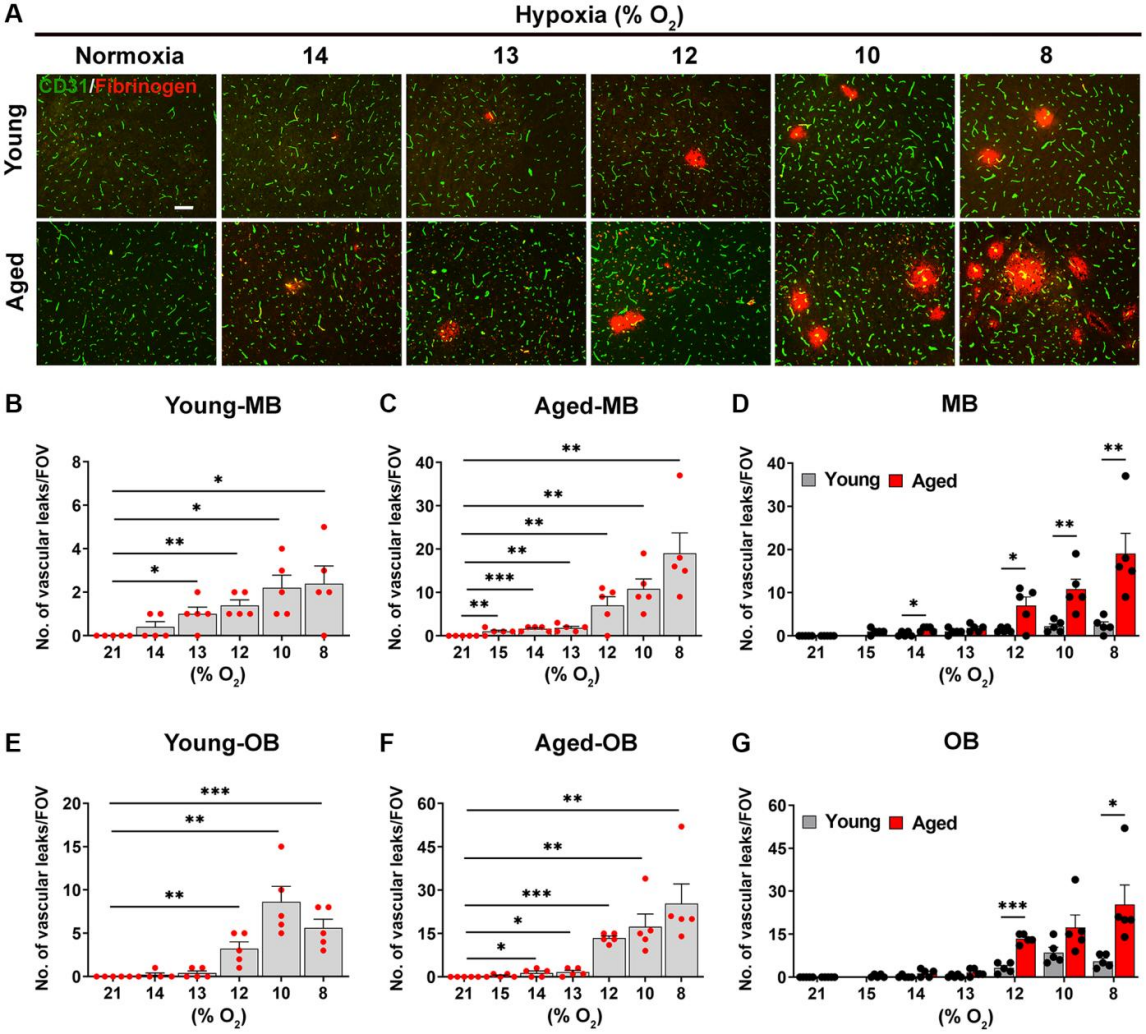
**Aged mice are more susceptible to hypoxia-induced BBB disruption.** (**A**) Frozen brain sections taken from young (2 months) and aged (20 months) mice exposed to normoxia or different levels of hypoxia (15–8% O_2_) for 4 days were stained for the endothelial marker CD31 (AlexaFluor-488) and fibrinogen (Cy-3). Images were captured in the midbrain. Scale bars = 100 μm. Quantification of the density of extravascular leaks following normoxia or 4 days exposure to the different levels of hypoxia in the midbrain (young (**B**), aged (**C**) and both combined (**D**)), and olfactory bulb (young (**E**), aged (**F**) and both combined (**G**)). Results are expressed as the mean ± standard error of the mean (SEM) (*n* = 5 mice/group). ^*^p < 0.05, ^**^p < 0.01, ^***^p < 0.001. One-way analysis of variance (ANOVA) followed by Tukey’s multiple comparison post-hoc test. Note that aged mice are more sensitive to the effects of hypoxia in that they display greater levels of BBB disruption at any given O_2_ level and they also display BBB disruption at a weaker hypoxic threshold than young mice.

Next, we compared young and aged mice for their susceptibility to hypoxia-induced BBB disruption by performing dual-IF with CD31 and fibrinogen, in which extravascular leak of fibrinogen indicates BBB breakdown. As shown in Figure 2A, no BBB disruption was seen under normoxic (control) conditions at either age, but as the hypoxic stimulus was increased (O_2._ level lowered), aged mice displayed a greater number of fibrinogen^+^ extravascular leaks, particularly in the hypoxic range 8–12% O_2._ The quantification revealed that in the midbrain, the hypoxic threshold that triggered significant BBB disruption, was 13% in young brain, but 15% in aged brain (Figure 2B, 2C). In a consistent manner, in the olfactory bulb, the hypoxic thresholds for young and aged brains were 12 and 14% O_2_, respectively (Figure 2E, 2F). It was also noticeable that at any level of hypoxia, the number of vascular leaks was always greater in the aged brain (Figure 2D, 2G).

### Microglia in the aged brain are more activated at every level of ambient O_2_

**Figure 3:**
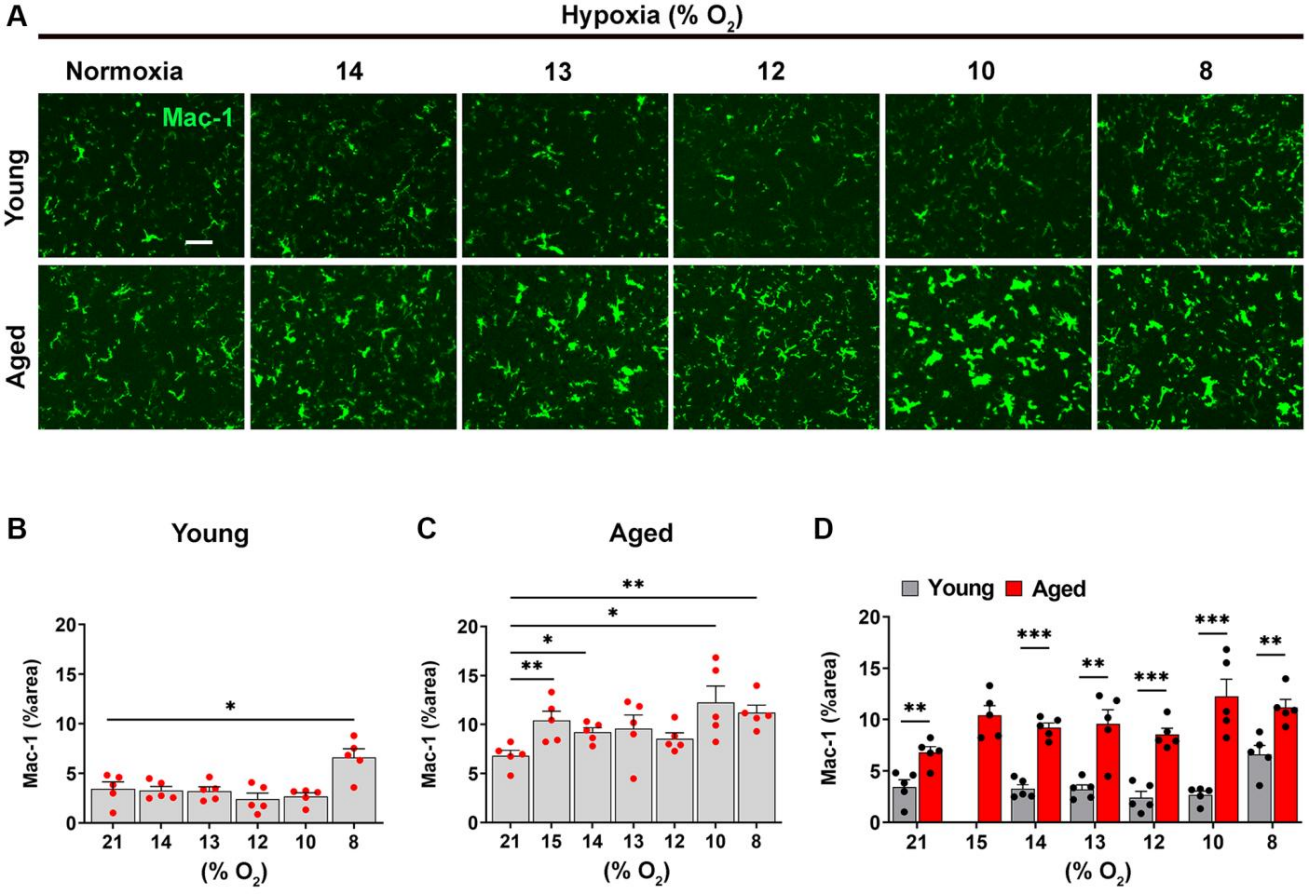
**Microglia in the aged brain are more activated at every level of ambient O_2_.** (**A**) Frozen brain sections taken from young (2 months) and aged (20 months) mice exposed to normoxia or different levels of hypoxia (15–8% O_2_) for 4 days were stained for Mac-1 (AlexaFluor-488). Images were captured in the midbrain. Scale bars = 100 μm. Quantification of the Mac-1 area following normoxia or 4 days exposure to the different levels of hypoxia in the midbrain in young (**B**), aged (**C**) and both combined (**D**). Results are expressed as the mean ± standard error of the mean (SEM) (*n* = 5 mice/group). ^*^p < 0.05, ^**^p < 0.01, ^***^p < 0.001. One-way analysis of variance (ANOVA) followed by Tukey’s multiple comparison post-hoc test. Note that microglia in the aged brain, were significantly more activated than those in young brain even under normoxic conditions, and in contrast to microglia in the young brain, the weakest hypoxia stimulus examined (15% O_2_) promoted significant increases in microglial activation compared to normoxic conditions.

As microglial activation is influenced by hypoxia and BBB disruption [2], we next examined the dose-response relationship between hypoxic dose and microglial activation in young and aged mice, using Mac-1 signal as an index (Figure 3). Consistent with previous findings, this showed that in the young brain, hypoxia had little impact on microglial activation, only stimulating significant increases in Mac-1 signal at the most severe level of hypoxia (8% O_2_; Figure 3A, 3B) [2]. By contrast, in the aged brain, microglia were significantly more activated than those in young brain even under normoxic conditions, and in contrast to microglia in the young brain, the weakest hypoxia stimulus examined (15% O_2_) promoted significant increases in microglial activation compared to normoxic conditions (Figure 3A, 3C). Interestingly though, when the level of hypoxic stimulus was further increased, it failed to enhance microglial activation over and above that seen at 15% O_2_. Of note, at any level of hypoxia, the level of microglial activation was always greater in the aged brain (Figure 3D). Taken together, these combined data demonstrate that the hypoxic threshold required to stimulate cerebrovascular remodeling is much weaker in the aged brain, or put another way, the aged brain is more sensitive to the effects of hypoxia.

### Defining the age at which the BBB becomes more susceptible to hypoxia

Having established that the aged brain is more vulnerable to BBB disruption following hypoxic exposure, this raises the important question: at what age does the BBB become more susceptible to hypoxia-induced disruption? Before embarking on these studies, we considered that the most likely outcome would be that the BBB would be relatively stable until middle age, i.e., 10–14 months old in mice, but then show signs of gradual deterioration that accelerate with age. To answer this question, we exposed female C57BL6/J mice of eight different ages (2, 4, 6, 9, 12, 15, 18, and 23 months) to a fixed (8% O_2_) level of hypoxia to determine at what age the BBB becomes more likely to undergo vascular remodeling and disruption due to hypoxic exposure. We evaluated endothelial proliferation, BBB disruption, microglial activation, and microglial proliferation, another sensitive index of microglial activation.

**Figure 4:**
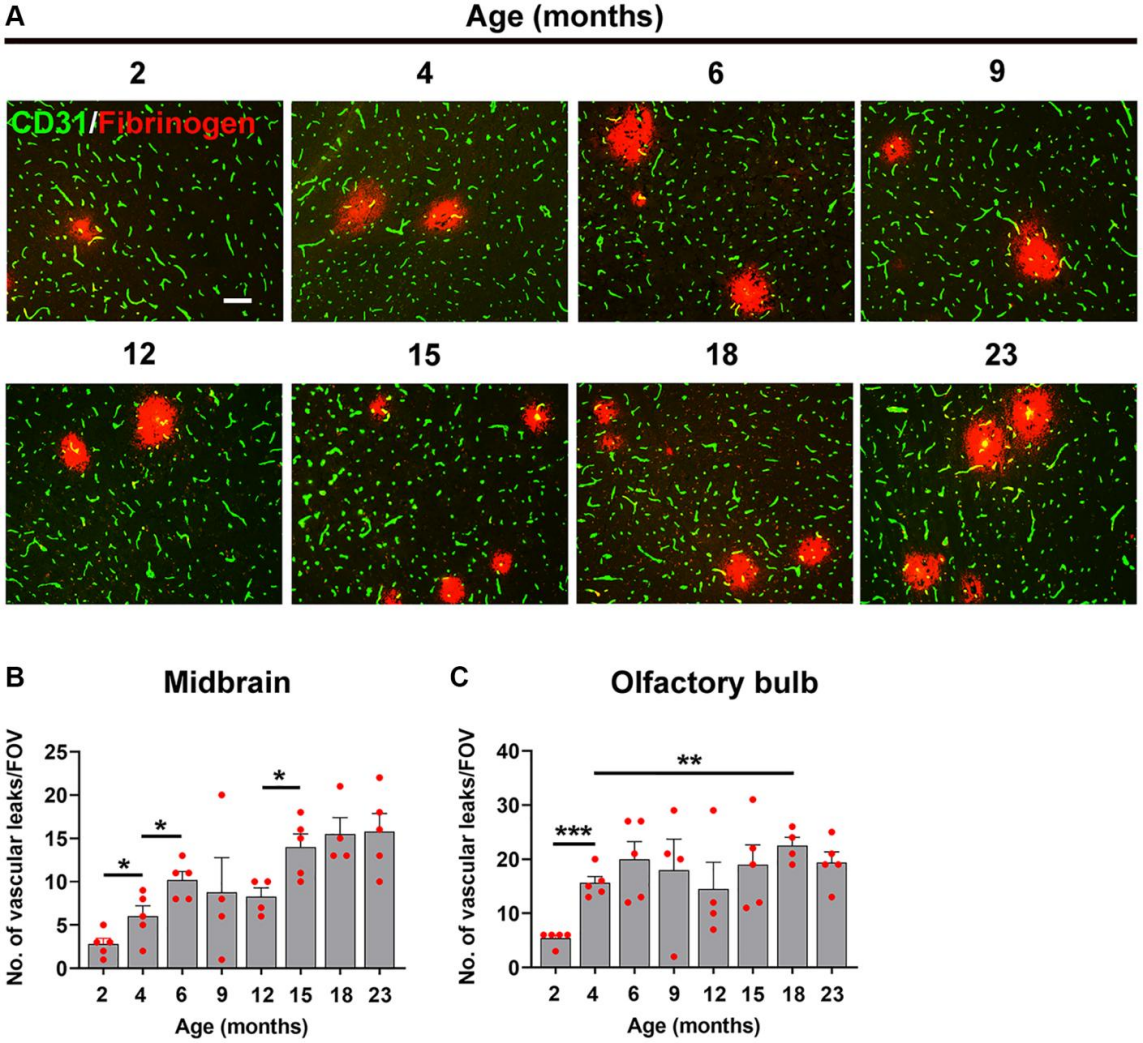
**Defining the age at which the BBB becomes more susceptible to hypoxia.** (**A**) Frozen brain sections taken from mice of different ages (2–23 months) exposed to hypoxia (8% O_2_) for 4 days were dual-stained for CD31 (AlexaFluor-488) and fibrinogen (Cy-3). Images were captured in the midbrain. Scale bars = 100 μm. Quantification of the density of extravascular leaks in the midbrain (**B**) or olfactory bulb (**C**) in different age mice following 4 days hypoxia. Results are expressed as the mean ± standard error of the mean (SEM) (*n* = 5 mice/group). ^*^p < 0.05, ^**^p < 0.01, ^***^p < 0.001. One-way analysis of variance (ANOVA) followed by Tukey’s multiple comparison post-hoc test. Note that the density of vascular leaks increased with age and that major increases were seen between 2–6 months, as well as between 12–15 months in the midbrain.

**Figure 5:**
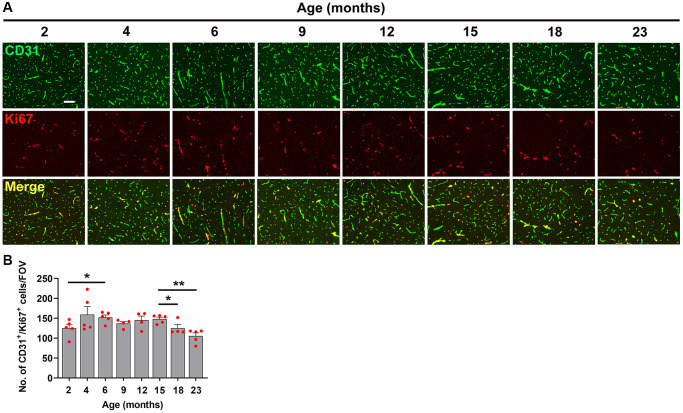
**Characterization of hypoxia-induced brain endothelial proliferation at different ages.** (**A**) Frozen brain sections taken from mice of different ages (2–23 months) exposed to hypoxia (8% O_2_) for 4 days were dual-stained for CD31 (AlexaFluor-488) and Ki67 (Cy-3). Images were captured in the midbrain. Scale bars = 100 μm. (**B**) Quantification of the density of CD31^+^/Ki67^+^ cells in the midbrain in different age mice following 4 days hypoxia. Results are expressed as the mean ± standard error of the mean (SEM) (*n* = 5 mice/group). ^*^p < 0.05, ^**^p < 0.01. One-way analysis of variance (ANOVA) followed by Tukey’s multiple comparison post-hoc test. Note that while endothelial proliferation was relatively constant across the age range, we observed a small but significant increase between 2–6 months and a small but significant decrease after 15 months.

As shown in Figure 4A, the density of fibrinogen^+^ extravascular leaks in the midbrain was very small in the youngest (2 month) mice, but gradually increased with age. Quantification revealed that the density of vascular leaks in the midbrain and olfactory bulb increase significantly with age (Figure 4B, 4C). When we examined at what age the increases occurred, we were surprised to see that the biggest step-up occurred between 2 and 6 months of age. This was consistent in both the midbrain (Figure 4B) and olfactory bulb (Figure 4C). In the midbrain, a further step-up occurred between 12 and 15 months, and the leak density then gradually plateaued up to the oldest age studied (23 months). The responses in olfactory bulb were similar in showing a big increase between 2 and 6 months but thereafter the vascular leak rate was maintained relatively constant right up to the oldest age studied. When we studied the endothelial proliferation rate by quantifying the number of CD31^+^/Ki67^+^ cells/FOV, we observed that all ages of mice showed a strong angiogenic response to 8% O_2_ (Figure 5A). Interestingly, when this was quantified, we observed that the rate of brain endothelial proliferation significantly increased between the ages of 2 to 6 months, then plateaued until 15 months, but then gradually declined thereafter to significantly lower levels at 18 and 23 months (Figure 5B). It is notable that the step-up in endothelial proliferation rate between 2 and 6 months coincides with a similar increase in the density of vascular leaks over the same time period, consistent with our previous finding that angiogenic responses and BBB disruption tend to show a close temporal correlation.

**Figure 6:**
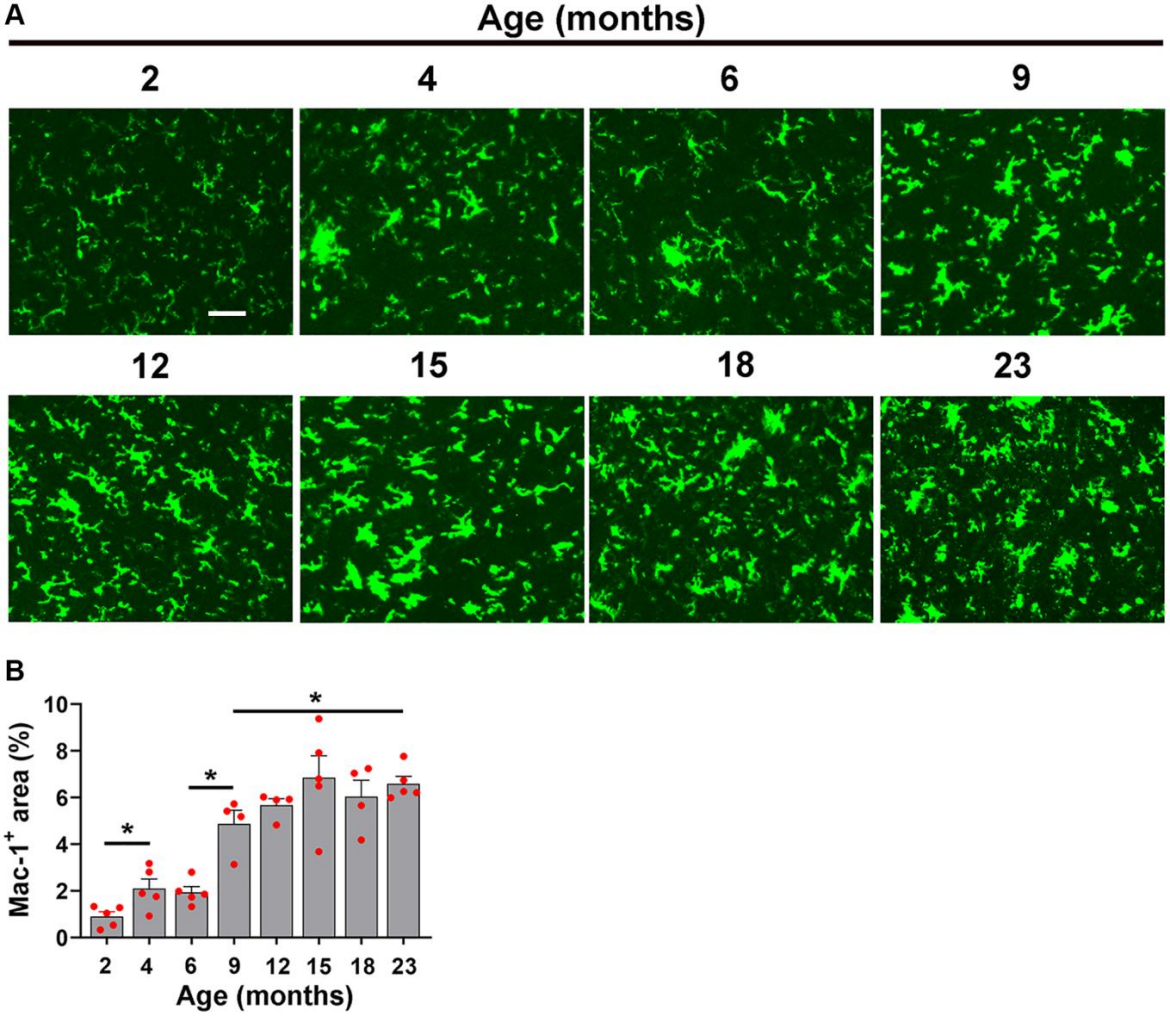
**Characterization of hypoxia-induced microglial activation at different ages.** (**A**) Frozen brain sections taken from mice of different ages (2–23 months) exposed to hypoxia (8% O_2_) for 4 days were labelled with Mac-1 (AlexaFluor-488). Images were captured in the midbrain. Scale bars = 100 μm. (**B**) Quantification of the Mac-1 area in the midbrain in different age mice following 4 days hypoxia. Results are expressed as the mean ± standard error of the mean (SEM) (*n* = 5 mice/group). ^*^p < 0.05. One-way analysis of variance (ANOVA) followed by Tukey’s multiple comparison post-hoc test. Note that the Mac-1 signal increased in a stepwise manner from 2 months and increasing steadily all the way up to 15 months, after which time it plateaued.

**Figure 7:**
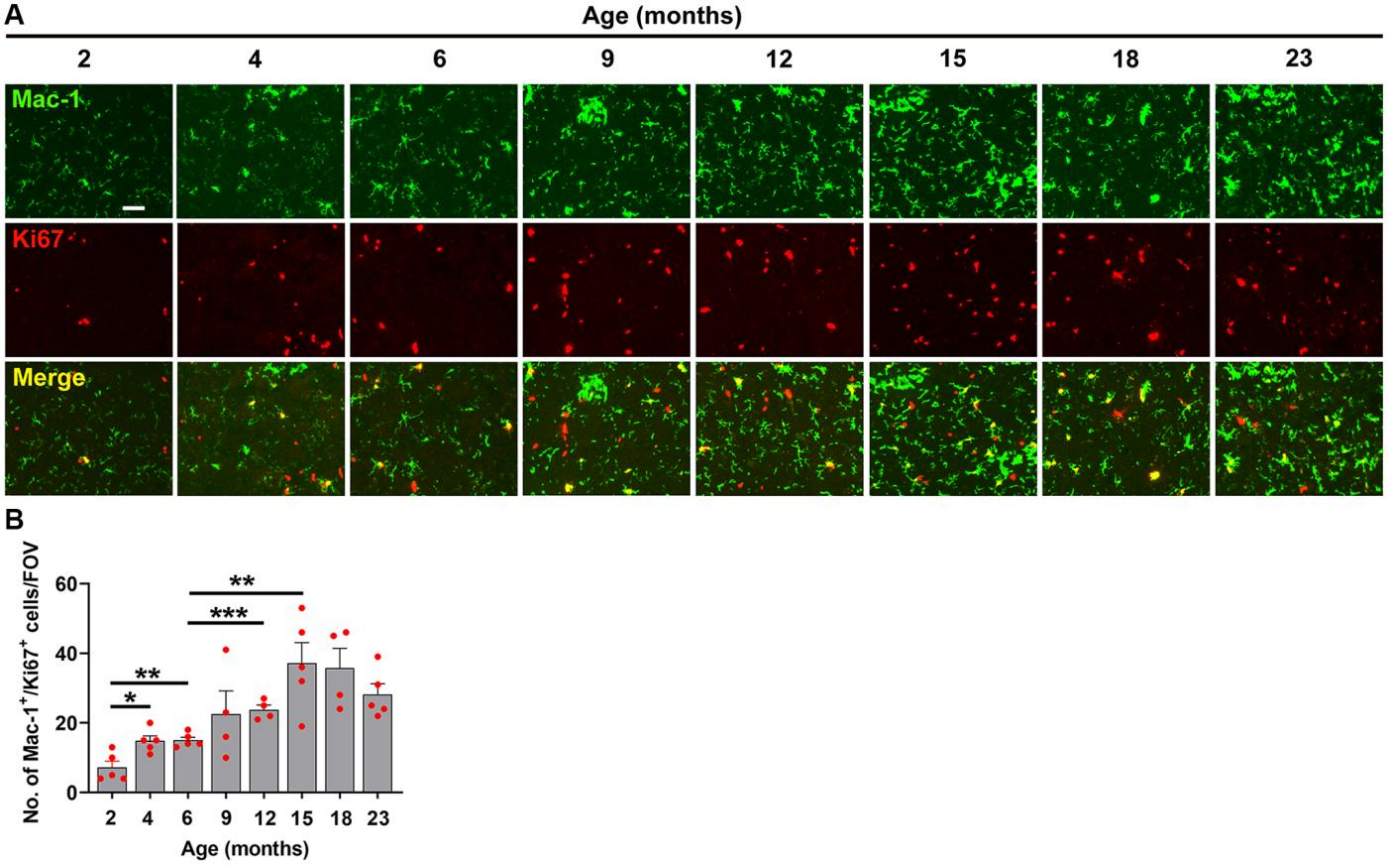
**Characterization of hypoxia-induced microglial proliferation at different ages.** (**A**) Frozen brain sections taken from mice of different ages (2–23 months) exposed to hypoxia (8% O_2_) for 4 days were dual-labelled with Mac-1 (AlexaFluor-488) and Ki67 (Cy-3). Images were captured in the midbrain. Scale bars = 100 μm. (**B**) Quantification of the Mac-1 area in the midbrain in different age mice following 4 days hypoxia. Results are expressed as the mean ± standard error of the mean (SEM) (*n* = 5 mice/group). ^*^p < 0.05, ^**^p < 0.01, ^***^p < 0.001. One-way analysis of variance (ANOVA) followed by Tukey’s multiple comparison post-hoc test. Note that microglial proliferation increased in a stepwise manner from 2 months and increased steadily all the way up to 15 months, after which time it plateaued.

When we studied the impact of hypoxia on microglial activation across the different age groups, we observed a slightly different type of kinetic response. Microglial activation as measured by total Mac-1 signal increased in a stepwise manner, starting at 2 months and increasing steadily all the way up to 15 months, after which it plateaued (Figure 6A, 6B). As microglial proliferation is another sensitive indicator of cellular activation, we also investigated this by quantifying the density of Mac-1^+^/Ki67^+^ cells/FOV. This revealed a kinetic pattern very similar to that of the Mac-1 signal, i.e., it steadily increased in a stepwise manner up to 15 months, then plateaued (Figure 7).

## DISCUSSION

Hypoxia triggers a strong adaptive vascular remodeling response in the brain that results in enhanced vascular density [9–11]. Of high translational significance, mice receiving hypoxic pre-conditioning are protected against subsequent cerebral ischemia, suggesting that this treatment triggers beneficial adaptive responses that better prepare the animal to resist stronger hypoxic insults [12, 13]. Interestingly, as part of this physiological adaptive response, transient BBB disruption also occurs [1, 2], which considering the importance of a tight BBB in the maintenance of brain homeostasis [14–16], could have pathological consequences. Hypoxia is common to many age-related diseases including chronic obstructive pulmonary disease (COPD), heart failure, and sleep apnea, and because hypoxia and many of these conditions predispose to neurodegeneration and cognitive decline [3–6], it becomes clinically important to define the hypoxic thresholds that trigger vascular remodeling and BBB disruption. To address this gap in knowledge, we designed this study to have two main goals. First, define the level of hypoxia that triggers cerebrovascular remodeling and determine if this is influenced by age. Second, using a fixed level of hypoxia (8% O_2_), define the age at which cerebral blood vessels become more susceptible to hypoxia-induced disruption. Our main findings were as follows: (i) aged mice were much more sensitive to hypoxia, meaning that hypoxia-induced cerebrovascular remodeling events (endothelial proliferation and BBB disruption) were first detected at a higher O_2_ level in aged (15% O_2_) compared to young (13% O_2_) mice, (ii) microglia in aged brain were significantly more activated than those in young brain even under normoxic conditions, and were activated further, even by the weakest hypoxia stimulus examined (15% O_2_), while microglia in young brain were only activated at the most severe hypoxic level examined (8% O_2_), (iii) though increased age had only minimal impact on the rate of hypoxia-induced endothelial proliferation, it strongly increased the degree of BBB disruption (4-6-fold greater in 23 months vs. 2 months old), (iv) age-related increases in hypoxia-induced BBB disruption occurred at a surprisingly young age, with a marked increase in hypoxia sensitivity occurring between 2 and 6 months, as well as a later jump between 12 and 15 months, and (v) levels of microglial activation in aged brain were at least 2-fold greater than those in young brain (even under normoxic conditions), as assessed by Mac-1 expression level, and overall, microglial activation and proliferation largely correlated with age-related increases in BBB disruption, increasing in a stepwise manner until 15 months of age, and then plateaued thereafter.

### The translational significance of defining hypoxic thresholds

Hypoxia is a common theme in many age-related diseases including chronic obstructive pulmonary disease (COPD), asthma, heart failure, ischemic heart disease, and sleep apnea [3–6]. Over a number of studies we have demonstrated a strong link between hypoxia and BBB breakdown, and more recently, between these events and neurodegeneration and cognitive decline [7]. Interestingly, several clinical studies have demonstrated an increased risk of dementia in people who suffer from hypoxia-inducing conditions such as sleep apnea and COPD [3–6]. Furthermore, anosmia (loss of smell) is recognized as an early prognostic indicator of dementia risk [17, 18]. As we have shown that the olfactory bulb is by far the most sensitive brain region to hypoxia-induced BBB disruption [1, 2], this implies that hypoxia-induced damage in this region may be a harbinger of things to come in relation to subsequent decline in cognitive function.

The strongest message to emerge from this study is that compared to young brain, the aged brain is far more susceptible to hypoxia-induced BBB disruption and associated microglial activation, as the hypoxia thresholds required to trigger vascular remodeling in young and aged mouse brain were defined as 13% O_2_ and 15% O_2_, respectively. From a clinical perspective, it is clearly optimal to maintain ambient O_2_ levels as close to that seen at sea level (21% O_2_). Our findings suggest that when ambient O_2_ levels drop below the critical thresholds for young and aged mice (13% O_2_ and 15% O_2_, respectively), some degree of vascular remodeling and BBB disruption will occur, which if sustained for long periods of time, could lead to neurodegeneration and cognitive decline. In addition to clinical conditions manifesting a hypoxic state, our findings also have direct relevance to mountaineers and hillwalkers. Oxygen levels of 13% O_2_ and 15% O_2_ equate to altitudes of approximately 12,400 and 8,600 ft, respectively. As our results indicate that age increases the sensitivity to hypoxic exposure, this suggests that mountain altitudes that were previously non-problematic for any given individual, may begin to trigger clinical symptoms of acute mountain sickness (AMS) and high-altitude cerebral edema (HACE) as people get older. However, in contrast to the findings we present here, epidemiological studies have so far failed to reach a consensus on whether age impacts the incidence or severity of AMS [19]. While some studies have shown that increased age increases AMS risk [20, 21], others have shown no clear relationship [22], and some have even suggested that age may be protective [23]. Potential reasons accounting for this inconsistency may include a multitude of factors including variability in the studied population (age range, sex, fitness level, previous mountain experience), severity of altitude challenge, or the speed of ascent. One caveat of our study is of course that mice are not human, and there are multiple incidences of findings in mice studies not being borne out in clinical studies, so it is possible that inter-species differences exist in BBB-regulatory mechanisms. However, most evidence suggests that cerebrovascular regulatory mechanisms are highly conserved between mice and humans. For instance, hypoxic exposure in humans also promotes increased cerebral blood flow [24, 25] and an adaptive angiogenic response that culminates in enhanced vascular density in the brains of high-altitude dwellers [26], just as it does in rodents [8, 10, 27]. It is important to consider that while these data define the hypoxic thresholds that trigger BBB disruption in young and aged mice, these thresholds may not be identical in humans; thus, further studies may be needed to establish these thresholds in humans. Furthermore, while our studies focused on BBB disruption in the mouse olfactory bulb and midbrain, other brain regions show far fewer leaks, demonstrating that the hypoxic thresholds that trigger BBB disruption differ markedly between different brain regions. Another interesting question to consider is does the form of hypoxia (disease versus high altitude exercise) have any impact or is the absolute level of O_2_ the primary driver of BBB breakdown? Based on what we know about vascular physiology, one might predict that any given level of hypoxia would cause more BBB disruption in a disease situation vs. a high-altitude exercise situation because of the increased likelihood that blood vessels in disease conditions are more likely to be inflamed and damaged while those in healthy individuals capable of undertaking high altitude extreme exercise are more likely to be strong and healthy.

In a recent study of aged mice, we addressed the fundamental question: do BBB disruptions repair when mice are switched back from hypoxic conditions to normoxia? This revealed that while blood vessels spontaneously repair after return to normoxic conditions, microglia are slow to return to their pre-hypoxic state and remain activated for an extended period of time [7]. The translational implication of this is that while disrupted cerebral blood vessels can repair, the persistently activated microglia may further amplify the neuroinflammatory state in the aged brain. One important factor we did not address in this study is sex-specific differences in these cerebrovascular responses. Recent studies show that with aging, women tend to maintain higher cerebral blood flow and show less capillary rarefaction than their male counterparts [28]. Furthermore, MRI studies using gadolinium enhancement have demonstrated that with aging, males show greater levels of vascular leak in the cerebral cortex, hippocampus, and white matter than females [29, 30]. Based on these findings, we predict that aged male mice might show greater levels of hypoxia-induced BBB disruption in our CMH model; we are currently investigating this possibility. While our studies were performed exclusively in the C57BL6/J strain of mice, it might also be insightful to repeat these studies in other strains, particularly the BALB/c strain. This is because BALB/c mice develop much bigger ischemic lesions in the middle cerebral artery occlusion (MCAo) models of ischemic stroke, which is thought to be a result of their limited ability to generate cerebral arterial branches and collaterals [31]. Interestingly, the LaManna lab showed that when these two strains are exposed to CMH, the C57BL6/J strain generates a much stronger angiogenic response than the BALB/c strain, which correlates with greater upregulation of the angiogenic factors vascular endothelial growth factor (VEGF), angiopoietin (Ang)-1 and Ang2, and the Ang receptor Tie2 [32].

### The relationship between endothelial proliferation and BBB disruption

In previous work we described that the extent of hypoxia-induced BBB disruption differs markedly between different brain regions, with high levels in the olfactory bulb and midbrain, but much lower in the cerebral cortex and cerebellum [1, 2]. Of note, where BBB disruption is high, so is endothelial proliferation which promoted us to wonder if vascular breakdown is simply an unwanted side-effect of the angiogenic process [1, 2]. However, in a subsequent study, we demonstrated that while the degrees of BBB disruption and endothelial proliferation correlate very strongly on a region-to-region basis, at the level of individual blood vessels, these events are very separate, with no co-localization between BBB disruption and endothelial proliferation [33]. Based on these observations, we concluded that endothelial proliferation is part of a healthy adaptive response in blood vessels, but that BBB disruption is a dysfunctional response that occurs in a distinct set of non-angiogenic blood vessels. Consistent with this, in the first part of the current study we noted a very tight correlation between endothelial proliferation and BBB disruption, with both being triggered together at the same hypoxic level, both in young (13% O_2_) and aged (15% O_2_) mice. However, in the second part of this study, we found that while the rate of endothelial proliferation showed only minimal variation across the full age range tested, levels of BBB disruption in 23-month-old mice were 6-fold greater than that in 2-month-old mice. From this we draw two inferences. First, it further supports the notion that hypoxia-induced endothelial proliferation and BBB disruption are not directly connected. Second, it suggests that the reason aged cerebral blood vessels leak 6-fold greater more than young is not because of a failure of the endothelial proliferative response, but more likely is due to a delay in a later phase of the angiogenic response, such that aged mice are slow to construct mature and stable cerebral blood vessels.

### Age-dependent changes in cerebrovascular remodeling

When we designed this study, we predicted that BBB stability might start to deteriorate sometime in middle age, which equates to ~10–14 months in the mouse. Our rationale was simple; this is the time of life when humans start to manifest neurological disease because of increased levels of atherosclerosis and inflammation due to the damaging effects of elevated blood pressure, glucose and cholesterol levels. In line with this expectation, there was a significant step-up in hypoxia-induced BBB disruption and microglial activation in the midbrain in the 12–15 months age range. However, unexpectedly, a large increase in BBB vulnerability to hypoxia also occurred between 2–4 months. This showed that young (2 months <) mice were relatively resistant to the effects of hypoxia, but soon after, hypoxia induced a much greater degree of BBB disruption and associated microglial activation. What could account for such a switch? We believe the most likely reason for this is that at ages less than 2 months, the brain is still at a highly plastic developmental stage that is more conducive to supporting a rapid and efficient vascular remodeling response to prevent BBB breakdown. However, within a few weeks, mice have moved beyond this developmental period and blood vessels have formed into mature vascular networks with reduced plasticity. Consequently, when 4-month-old mice meet a hypoxic challenge, the more mature blood vessels are slower to mount a rapid remodeling response, resulting in greater levels of BBB disruption. Alternatively, this sudden decline in BBB integrity at such an early age (2–4 months) may be a result of reduced expression, organization, or phosphorylation of specific tight junction proteins such as claudin-5, occludin, or ZO-1. In follow up studies, we will examine this more closely.

## CONCLUSIONS

Our data define the hypoxic thresholds that trigger cerebrovascular remodeling in young and aged mice, thereby demonstrating that blood vessels in aged brain are far more vulnerable to hypoxia-induced BBB disruption than young. This increased BBB vulnerability is associated with greatly enhanced microglial activation. Our data also define the ages when the BBB becomes more sensitive to hypoxia, showing increased susceptibility between 12–15 months old but also surprisingly at the earlier 2–6 months age range. As advanced age is associated with increased incidence of hypoxia-associated conditions such as asthma, emphysema, ischemic heart disease, heart failure, and apnea, our findings have important implications for many people. It thus becomes an important priority to generate novel therapeutic strategies that can either delay age-related BBB deterioration or enhance BBB integrity in the hypoxia-susceptible elderly population.

## MATERIALS AND METHODS

### Animals

The studies described were reviewed and approved by the Institutional Animal Care and Use Committee at San Diego Biomedical Research Institute (SDBRI; protocol number 21-0006-2). Female C57BL6/J mice of all ages were obtained from Jackson Laboratories and the NIH National Institute on Aging rodent colony and were maintained under pathogen-free conditions in the closed breeding colony of SDBRI.

### Chronic hypoxia model

In the first set of studies, young (2 months old) and aged (20 months old) female C57BL6/J mice were exposed to a range of oxygen levels including 21% (normoxia) and 15, 14, 13, 12, 10, and 8% O_2_. To create hypoxic conditions, mice were housed four to a cage and placed into a hypoxia chamber (Oxycycler A-series (A84), Biospherix, Redfield, NY, USA) maintained at the level of oxygen determined by the user. In this system, atmospheric oxygen level is regulated via an in-chamber oxygen sensor, a control module for regulating gas flow, and a computer with control and analysis software. This system creates a desired level of oxygen at atmospheric (normobaric) pressure and is finely tuned to maintain the desired oxygen tension for the duration of the experiment to mimic high altitude atmospheric conditions. In the second set of studies, we exposed female C57BL6/J mice of 8 different ages (from 2 months to 23 months) to a fixed (8% O_2_) level of hypoxia. Every two days, the chambers were briefly opened for cage cleaning and food and water replacement as needed. In both studies, brain tissue was harvested after 4 days hypoxic exposure for histological analysis.

### Immunohistochemistry and antibodies

Immunohistochemistry was performed on 10 µm frozen sections of cold phosphate buffer saline (PBS) perfused tissues as described previously [34, 35]. Briefly, at the termination of the study, mice were subject to deep anesthesia using a cocktail of ketamine and xylazine and transcardially perfused. Rat monoclonal antibodies from BD Pharmingen (La Jolla, CA, USA) reactive for the following antigens were used in this study: CD31 (clone MEC13.3; 1:300), and Mac-1 (clone M1/70; 1:50). Rabbit polyclonal antibodies reactive for fibrinogen (1:2000; Millipore, Temecula, CA, USA ) or Ki67 (1:4000; Novus Biologicals, Centennial, CO, USA) were also used. Secondary antibodies used include Cy3-conjugated anti-rabbit (1:1000; Jackson Immunoresearch, West Grove, PA, USA) and Alexa Fluor 488-conjugated anti-rat (1:500; Invitrogen, Carlsbad, CA, USA).

### Image analysis

Images were taken using an Axioskop2 plus microscope (Carl Zeiss, Dublin, CA, USA) equipped with an Infinity 3S camera (Lumenera, Ottawa, ON, Canada) and Infinity Analyze imaging software (Lumenera). For each antigen in all analyses, images of at least three randomly selected areas were taken at 5×, 10× or 20× magnification per tissue section and three sections per brain analyzed to calculate the mean for each animal (*n* = 5 mice per group). The number of vascular leaks and proliferating endothelial cells or microglia per field of view (FOV) was quantified by capturing images and performing manual counts. Total Mac-1 fluorescent signal per FOV was measured and analyzed using NIH ImageJ Software.

### Statistical analysis

Each experiment was performed with 5 different animals per condition, and the results expressed as the mean ± SEM. Statistical significance was assessed using one-way analysis of variance (ANOVA) followed by Tukey’s multiple comparison post-hoc test, in which *p* < 0.05 was defined as statistically significant.

### Availability of data and material

The datasets used and/or analysed during the current study are available from the corresponding author on reasonable request.
